# Photoelasticity for Stress Concentration Analysis in Dentistry and Medicine

**DOI:** 10.3390/ma15196819

**Published:** 2022-09-30

**Authors:** Miriam Marín-Miranda, Ana María Wintergerst, Yoshamin Abnoba Moreno-Vargas, María Lilia Adriana Juárez-López, Cesar Tavera-Ruiz

**Affiliations:** 1 Facultad de Estudios Superiores Zaragoza, UNAM, Mexico City C.P. 09230, Mexico; 2 Facultad de Odontologia, UNAM, Mexico City C.P. 04510, Mexico; 3 Universidad de Monterrey, San Pedro Garza García C.P. 66238, Mexico

**Keywords:** biomechanics, didactics, photoelastic models, photoelasticity, stress concentration

## Abstract

Complex stresses are created or applied as part of medical and dental treatments, which are linked to the achievement of treatment goals and favorable prognosis. Photoelasticity is an optical technique that can help observe and understand biomechanics, which is essential for planning, evaluation and treatment in health professions. The objective of this project was to review the existing information on the use of photoelasticity in medicine and dentistry and determine their purpose, the areas or treatments for which it was used, models used as well as to identify areas of opportunity for the application of the technique and the generation of new models. A literature review was carried out to identify publications in dentistry and medicine in which photoelasticity was used as an experimental method. The databases used were: Sciencedirect, PubMed, Scopus, Ovid, Springer, EBSCO, Wiley, Lilacs, Medigraphic Artemisa and SciELO. Duplicate and incomplete articles were eliminated, obtaining 84 articles published between 2000 and 2019 for analysis. In dentistry, ten subdisciplines were found in which photoelasticity was used; those related to implants for fixed prostheses were the most abundant. In medicine, orthopedic research predominates; and its application is not limited to hard tissues. No reports were found on the use of photoelastic models as a teaching aid in either medicine or dentistry. Photoelasticity has been widely used in the context of research where it has limitations due to the characteristics of the results provided by the technique, there is no evidence of use in the health area to exploit its application in learning biomechanics; on the other hand there is little development in models that faithfully represent the anatomy and characteristics of the different tissues of the human body, which opens the opportunity to take up the qualitative results offered by the technique to transpolate it to an application and clinical learning.

## 1. Introduction

The principles of physics and mechanics are applied in the daily practice of medicine and dentistry, although this is almost never done consciously, probably because few teaching methods during training reveal their relevance. The application of these concepts to biological systems is referred to as biomechanics (i.e., the distribution of forces on different parts of the body) [1]. Biomechanics, for example, can explain that the reason why most fractures after a strong blow to the chin take place in the mandibular angle, is that stress concentrates around holes, defects and geometrical irregularities [2]. Bones and teeth have a complex geometry since they have to distribute forces generated during function such as during chewing [3]. Oral diseases (e.g., caries, malocclusion, cancer) as well as treatments (e.g., restorations, prostheses, orthodontics) modify the form of dental and bony structures and even if our bodies try to adapt to these changes, muscular chains, bones or joints may be overwhelmed triggering pain, bone remodelling, or muscular or joint lesions [4].

It is therefore important to understand biomechanics to identify treatments that may initiate injuries or to be able to devise treatments that could restore equilibrium. We therefore need models that represent what happens clinically which will thus help in attaining knowledge. It is difficult to study biomechanics directly on biological systems, and it is therefore necessary to rely on simulations. Simulation techniques allow the analysis of phenomena without the need to work on the object of analysis itself. Some of the methods that have been used to analyze stress concentration include electric extensiometry [5], finite element modeling [6,7,8], interferometry [9] and Tresca stress study [10], but these methods are somewhat complex requiring specialized equipment/technology [11].

Another technique that has been applied in combination with these is photoelasticity, which allows analyzing the stress distribution in birefringent materials subjected to mechanical loads by observing the fringe pattern (isochromatic or isoclinic) produced by the optical phase differences of light passing along the material under study [12]. For isotropic optical materials, mechanical stress will lead to structure deformation, resulting in local density differences along the material axes and the change of the refractive index; then, when the light beam passes through those materials birefringence will occur. According to the plane stress-optic law [13], the stress and the refractive index can be described in a plane perpendicular to the direction of light propagation as follows:

(1) $$n_{e}-n_{o}=K.\left(\sigma_{1}-\sigma_{2}\right)$$

where $n_{e}$ and $n_{o}$ correspond to the refractive index along the diffraction direction of extraordinary (refracted) and ordinary (non-refracted) light, respectively; $\sigma_{1}$ and $\sigma_{2}$ are the first and second principal stresses, respectively, and K is the photoelastic coefficient of the material. When a linear polarized light is perpendicularly incident to the material sample with thickness d, the light vector can be represented into two vectors in the perpendicular plane to the direction of its propagation, which vibrates along and perpendicular to the direction of principal stress. By the birefringence effect, the optical path difference Δ of the two linear polarized lights after passing through the sample can be expressed as:

(2) $$\Delta =d.\left(n_{e}-n_{o}\right)$$

and

(3) $$\left(\sigma_{1}-\sigma_{2}\right)=\frac{\Delta }{K.d}$$

is derived from Equations (1) and (2) and conforms the relationship between stress and the optical path difference Δ. The left side in Equation (3) helps to explain that stress must be understood as a relative measurement which needs a previous (accumulated) and a current stress status in the sample under analysis.

By representing the optical path difference by its length terms where Δ = *δ*.(*λ*/2*π*) stress can also be described as length quantity as:

(4) $$\left(\sigma_{1}-\sigma_{2}\right)=\frac{\delta }{K.d.2\pi }.\lambda$$

where δ is the phase difference in radians and λ represents the light wavelength (nm). The quotient Δ/d (nm/cm) is called the birefringence retardation and refers to the optical path delay (nm) after passing through a sample with a certain thickness (cm). From Equations (3) and (4) we can observe that the birefringence retardation is proportional to the principal stress difference. Finally, the stress can be calculated according to the optical path difference or phase difference considering the sample thickness and the photoelastic coefficient [14,15].

Given that photoelasticity contributes to the understanding of force distribution and concentration in simulation models, this technique has been used experimentally to study areas of stress concentration of medical and dental treatments. Reports on the use of this technique in these disciplines have been published in the scientific literature since the 1950s, contributing to important changes or innovations of treatments and materials in the area [16,17,18,19].

Academic programs of the schools of medicine and dentistry include applied biomechanics and anatomical simulation models to teach the location, shape and connections of organs and tissues, for example, the origin and insertion of a muscle. Simulation models aim to faithfully reproduce the conditions of the model they intend to simulate; however, the musculoskeletal system and human anatomy are too complex to be mimicked. How well do the models reflect this complexity? How much do these models consider the different tissues in the area that is studied?

The use of models or simulations improves the quality of learning [20]. In engineering careers, photoelastic models are used to help students identify areas where structures can fail [21]. Identifying existing models and how they have been used could provide the basis for the application of these techniques as novel didactic models that could favor a better understanding of biomechanics by students in the health area. The objective of this project was to review the existing information on the use of photoelasticity in medicine and dentistry and determine their purpose, the areas or treatments for which it was used, models used, as well as to identify areas of opportunity for the application of the technique and the generation of new models.

## 2. Materials and Methods

### Sources of Information and Search Strategies

For this review we searched nine electronic databases: Sciencedirect, PubMed, Scopus, Springer, EBSCO, Wiley, Lilacs, Artemisa and SciELO; we additionally searched Google Scholar and web pages of universities and related associations. The following keywords were used: photoelasticity, photoelastic, biomechanics, “stress concentration”, “stress concentration analysis”, “bone model”, “dental model” and “periodontal models”, in different combinations using Boolean operators (AND/OR). The search focused on scientific articles, as well as theses, congress reports, and popular science articles related to the application of photoelasticity for stress concentration analysis in the dental and medical area; the documents selected were in English, Spanish and Portuguese.

**Figure 1:**
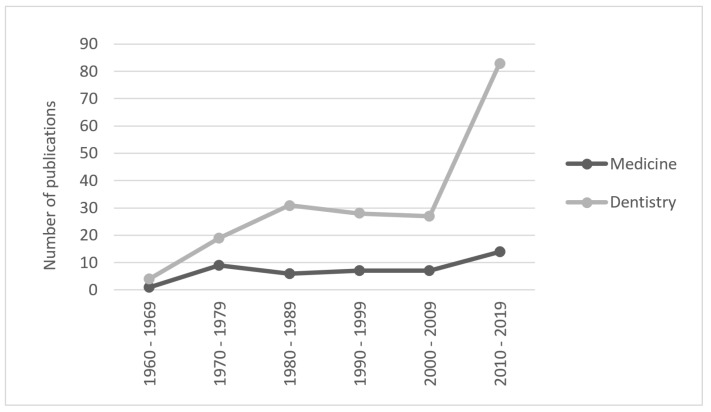
Number of publications in dentistry and medicine.

**Figure 2:**
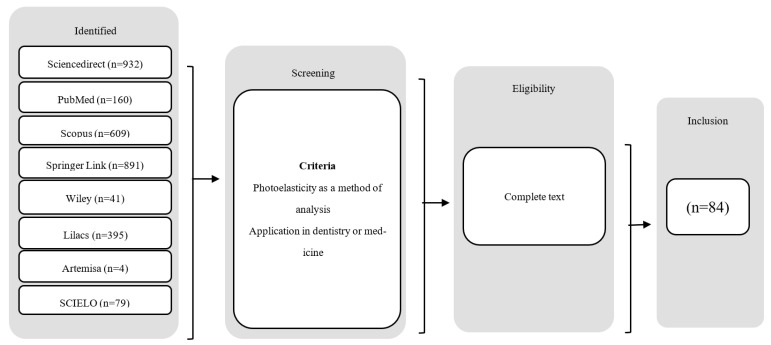
Selection process of published papers.

We identified 192 documents (1964–2019) and found a greater increase in publications on the subject since 2000 especially in the dental field (Figure 1) probably due to the development of the technology and materials that allowed the overcoming of previous limitations. In 2003, Orr [22] described the evolution of the technique and its application in the study of biomechanics; he mentions the importance of the development of new materials and the addition of quantitative analysis to improve the applications in medicine. We therefore decided to only include articles 2000–2019 so that the protocols did not differ dramatically in relation to the technique. Articles with full text where photoelasticity was applied as an experimental or analytical method and with direct application in dentistry or medicine were selected. Documents found were classified into dentistry or medicine, and subclassified by disciplines (Figure 2).

## 3. Results

**Figure 3:**
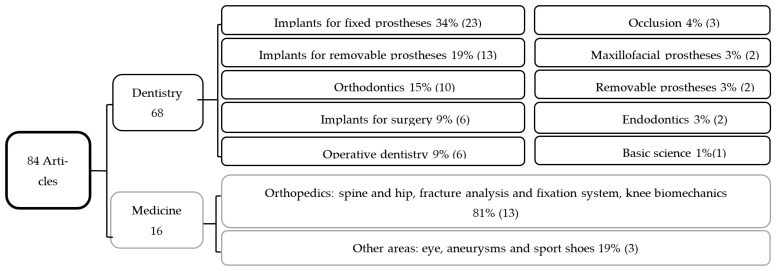
Distribution of articles by area of application.

In the dental field, 87 documents were found (2000 to 2019): 68 scientific articles, 16 theses, two conference reports and one dissemination article. Photoelasticity was applied in 10 different areas (Figure 3); most papers related to implants for fixed prostheses (34%), implants for removable prostheses (19%), and orthodontics (15%). Twenty documents were identified in the medical field: 16 scientific articles, one book chapter and three theses. The area of greatest impact in medicine is orthopedics (81%), where they have analyzed stress concentration in both hard and soft tissues (Figure 3).

The articles found in both dentistry and medicine are described, ordered from the area of greatest to least coverage (Table 1, Table 2, Table 3, Table 4, Table 5, Table 6, Table 7, Table 8, Table 9, Table 10, Table 11 and Table 12). It is important to mention that some articles included models with bone structures separated from dental and/or periodontal structures; these are more complete models and are marked in bold in the tables.

Next, Articles with application in the medical area are described.

**Table 11:** Characteristics and findings of studies on orthopedics in the medical area.

Reference	Objective	Type of Model and Photoelastic Material Used	Data
Plath et al., 2000 [89] Arch. Orthop. Trauma. Surg. Germany	To study reasons for the failure of custom-made stems manufactured according to biplanar X-rays.	Femur included in resin. Transparent polycarbonate.	Stress areas susceptible to failure in femoral prostheses.
Hirokawa et al., 2001 [90] J. Biomech. Japan	To understand how strain distributions along the fiber bundles of the anterior cruciate ligament change with knee motion.	Resin coated corpse knee. Polyurethane film.	Changes in stress distribution under different angles of movement of the knee.
Orr 2003 [22] Ir. J. Med. Sci. North Ireland	To describe the history of three-dimensional model analyses and methods for the photoelastic study of cancellous bone.	Reports on models reproduced in phenol formaldehyde and epoxy resin.	Demonstrates the utility of photoelasticity for biomechanical analysis.
Papachristou 2004 [91] Arch. Orthop. Trauma. Surg. Greece	Observation of the direction of the significant contact and internal stresses of the knee joint at every point in a single plane.	Reproduction of a knee in resin. Araldite^®^ by Huntsman, Brazil epoxy resin.	Patterns of isochromatics, isoclinics and trajectories of the knee joint.
Murphy et al., 2005 [92] J. Biomech. Ireland	To determine if load sharing occurs between the acromion and glenoid so as to reduce the high stresses experienced in the cement mantle relative to a prosthesis without acromion-fixation.	Scapula model in photoelastic resin. PL-8 PL-1 Vishay Measurements Group Inc., Group UK.	Stress distribution generated by glenoid components fixed to both the glenoid and acromion.
Siqueira et al., 2009 [93] Acta Orthop. Bras. Brazil	To analyze internal tensions near the medullar canal of photoelastic vertebra models using different screw sizes of the vertebral fixation system submitted to pullout strength.	Reproduction of vertebra in resin. Polipox epoxy resin. Sao Paulo, Brazil.	Internal tensions near the medullary canal in vertebrae models that use different screw sizes from the spinal fixation system subjected to extraction force.
Ellenrieder et al., 2012 [94] J. Orthop. Sci. Germany	To compare patterns of femoral cortical tension, before and after an implant under conditions of load and muscular strength.	Resin coated femoral prosthesis. PL 1 Vishay Measurements Group Inc., Raleigh, NC, USA.	Strain pattern in cases of femoral bone defects when placing a load on the distal interlocking screws.
Fakhouri et al., Acta Orthop. Bras. 2012 [95] Brazil	To analyze the shear forces on the vertebral body L4 when submitted to a compression force.	Simulation of sagittal vertebra in photoelastic resin. Polipox flexible epoxy resin. Sao Paulo, Brazil.	Stress generated in the lower lumbar area due to compression.
Stein et al., Surg. Radiol. Anat. 2012 [96] Germany	To study the biomechanical effects of axial weight-bearing on the stability of the fixed tibiofibular syndesmosis and the mortise in the treatment of ankle injuries.	Joint representation in resin molds. Not specified.	Force distribution through the screw into the cranial and caudal parts of the distal fibula.
Francés et al., 2013 [97] Musculoskelet. Surg. Spain	To study and validate the mechanical behavior of the bone-implant total hip prosthesis and the treatment of its complications.	Reproduction of a femur by 3D printing in resin Polycarbonate.	Residual tensions caused by local contact of the internal wall of the femur with the end of the stem.
Fakhouri et al., Acta Orthop. Bras. 2014 [98] Brazil	To compare internal stress caused by different vertebral fixation screws, when submitted to three different pullout strengths.	Bone fixation elements included in a resin mold. Polipox flexible epoxy resin. Sao Paulo, Brazil.	Internal shear stress produced by USS II type screw with external diameters of 5.2 and 6.2 mm.
Rodríguez-Cañizo et al., 2016 [99] Rev. Fac. Ing. Univ. Antioq. Mexico	To study the effect of annular fiber injuries in intervertebral discs as a result of trauma, evaluating the structural integrity of the whole system.	Human vertebrae covered by photoelastic material. PL 1 Vishay Measurements Group Inc., Raleigh, NC, USA.	Stresses in different areas of the vertebral body of L3.
Ye et al., 2016 [100] Springer Verlag China	To analyze new surgical treatments for children with scoliosis during growth.	Vertebral simulation. Not specified.	Stress on vertebrae under different corrective loads along the spine.

**Table 12:** Characteristics and findings of studies in other areas of application in medicine.

Reference	Objective	Type of Model and Photoelastic Material Used	Data
Driscoll et al., 2010 [101] Procedia Eng. UK	To identify surface shear stresses during running.	Resin plate. Not specified.	Maximum shear stress that occurs when the sole comes into contact with the photoelastic surface.
Doyle et al., 2012 [102] J. Biomech. Ireland	Assessment of wall strain in aortic aneurysms with complex specific geometries.	Reproduction of aneurysms in resin. PL-3 epoxy resin, PC-11 coating Vishay Measurements Group Inc., Raleigh, NC, USA.	Use of the photoelastic method to assess wall strain and its validation using finite element analysis.
Falconer et al., 2019 [103] Mater. Today: Proceedings UK	To develop a soft tissue surrogate.	Experimental polymer created to replace skin.	Mechanical properties of a gel to replace the skin.

## 4. Discussion

### 4.1. Dentistry

The results show greater application in the field of fixed and removable prostheses associated mostly with the use of implants (Table 1 and Table 2), which agrees with the findings of previous reviews [104], probably because failures due to rejection were previously associated with the load that the implants receive. Photoelasticity has provided the possibility of analysis and understanding of the phenomena of distribution of efforts to contribute to designs that generate less probability of rejection. Recent studies continue comparing different designs and variations in implantology techniques which suggests that there is still much to contribute towards better treatments [105,106,107,108] and some combine photoelasticity with other techniques such as finite element analysis providing a better description of the results [109].

**Figure 4:**
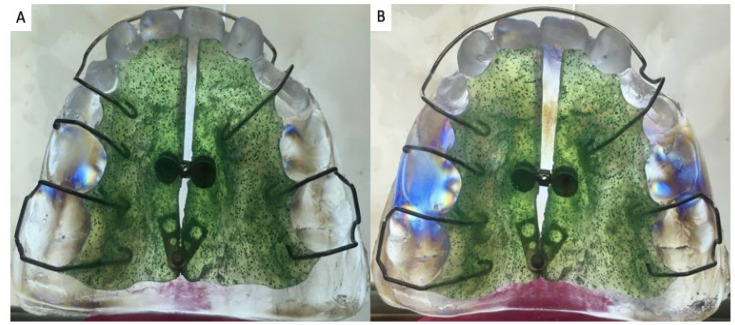
Example of the use of photoelasticity in dentistry with the placement of an expansion device on a photoelastic model of a patient. (**A**) Sitting of the appliance without activation of the screw; the isochromes indicate that the appliance is not passive and that stresses are already present due to (1) a poor hook design (2) an irregular vestibular archwire and (3) the presence of cavities due to caries. (**B**) After activation of the screw (observe the opening of the midline), the isochromes intensify in the areas where they were seen before and also spread towards the palate [110].

The application of the photoelastic technique in orthodontics was also found, since applied loads and the phenomena related to the modification of bone and dental biological structures are due to the applied forces (Table 3). Figure 4 shows dental students how an orthodontic appliance works and how its design as well as the characteristics of the patient’s teeth influence the areas where stress concentrates. These models can help students to analyze the importance of the design of appliances and the areas that could be damaged if the treatments are not adequately implemented or activated.

Other areas such as operative dentistry and occlusion in which biomechanics is central have just begun to take advantage of this technique; the number of papers found is increasing (Table 4, Table 5, Table 6, Table 7, Table 8, Table 9 and Table 10). The use of photoelastic models has generated important contributions in different areas of dentistry, defining the attachments and techniques that are most favorable considering the generation of efforts. However, they use different models, limiting photoelastic models according to their specific interest. It is noteworthy that only some take the general anatomy into account, and few consider the different properties of the resins. Some have made efforts to reproduce the characteristics of tissues that have very different strength and hardness; only six of the articles marked in bold considered these differences. It is necessary to develop models that come closer to clinical reality, considering the complexity of the anatomy and the importance of masticatory loads.

Some papers mention photoelasticity as an accessible technique for analysis for a specific purpose, such as Al-Omiri et al. [111] where they review the biomechanical behavior of teeth restored with endoposts with the possibility of making recommendations on techniques and materials that provide better clinical results. They mention how methods such as photoelasticity and finite element analysis back recommendations by providing important information, which supports our proposal for the use of photoelasticity as a didactic tool to demonstrate the importance of biomechanics to dentists.

Although there are other techniques beside photoelasticity that may facilitate learning biomechanics, most are complex and specialized (e.g., finite element analysis, interferometry), that may explain why we did not find any study using these techniques to explain biomechanics to dental students. It would probably make the understanding of biomechanics even more difficult because they would also have to understand the operational basis behind these other techniques. Even if it is easier to visualize stress with photoelasticity, we did not find any mention of its use either as a teaching aid in dentistry.

While there is a previous review on photoelasticity in dentistry [104], the review concentrated on the experimental use, focusing on the numerical validation of the method, probably due to the importance of comparing materials and techniques. Although they do mention that this technique is very useful because of the geometry of the models, they do not specifically mention their use as a teaching aid.

### 4.2. Medicine

There are few papers related to the application of photoelasticity in medicine although there are many bibliographic reference books concerning applied biomechanics in medical education. For this reason, in the medical area biomechanics is used to teach how fractures, dislocations and other traumas are induced and how different parts of the body work; mostly, with anatomical models, figures and diagrams with applied forces [112], but we did not find any documents mentioning photoelasticity for teaching.

Although we found fewer papers on photoelasticity in the medical area, the importance of biomechanical principles is better emphasized. Photoelasticity facilitates its understanding and, as in dentistry, its main objective is to improve devices and techniques, as well as an in-depth analysis of the causes that generate pathologies, fractures or affect the prognosis of treatments. The area that has exploited this technique either on its own or in combination with others, such as finite element, is orthopedics (Table 11). Prosthetic treatments and the knowledge of how different pathologies or traumatic injuries are produced has been extended thanks to the study of human biomechanics. In orthopedic surgery the photoelastic technique has contributed analyzing procedures with spinal and hip implants among others, as well as other surgical procedures (Table 12). It is important to point out that some studies do not only include bone elements but also joint and muscle elements, broadening the perspectives in the application of the technique. In other words, in medicine this technique has not been limited to the study of hard tissues of the human body.

In more recent papers in medicine the application goes beyond the analysis of forces on the body. For example, Maxwell et al. (2020) studied a dynamic image of photoelasticity to analyze ultrasound waves used to rupture urinary tract stones [113]. In medicine, improvements of different techniques that could be used together with photoleasticity are being developed [114].

A point to highlight in the medical area is that the type of models that could be developed may be more complex and diverse, due to the number of components, the different types of joints and the type of movements the joints perform, while in dentistry there are only two types of joints: the temporomandibular and the alveolo-dental, with the same models replicated in different publications.

### 4.3. Compiled Considerations

The results of this review indicate that photoelasticity has been used in different fields of medicine and dentistry. In dentistry most studies relate to implants for fixed and removable prostheses, and some have applied this technique to better understand materials or techniques for direct or indirect restorations, orthopedic and orthodontic appliances, as well as surgical or therapeutic techniques. Finding only 68 papers in dentistry and 16 in medicine during the period between 2000–2019 indicates that this technique has been underused.

The distribution of the reports shown in Figure 1 may be due to the development and advances in methods to obtain and process the data, and the improvement in the techniques as well as improvements in the production of the models. As an example of the improvements, we found the use of mineral oil to smoothen the change in the refraction indexes allowing a better signal from the isochromes [106,115], as well as the standardization in the thickness of the samples and stress-freezing [18].

Photoelasticity, similar to other techniques, offers advantages and disadvantages in relation to the precision of the results depending on the type of models used. Photoelasticity is an experimental technique that, despite not being a numerical technique by itself, is nevertheless valuable since it can back other techniques to obtain better results of the application to biological systems with complex geometries. Although photoelasticity is better adjusted to flat 2D models for the observation of the isochromes, it has been adapted to be used in 3D models or thicker ones. Of course, based on the required result there is always the possibility of complementing some techniques with others. Photoelasticity, however, has merits that grant its validity with the possibility of obtaining visual results to observe stress and strain in complex geometries that are more difficult to observe with other techniques [18,68,104] and that if needed can provide numerical values.

An important finding was that most studies used simple models and that complex anatomy is not considered, reproducing only the small area of interest for the device under study (e.g., a dental implant in a cube of resin [28] or a reproduction of the alveolar process with dental implants [55]). A slightly more complex model includes individual teeth in one type of resin enclosed in a maxillary section made out of another type of resin [43].

Of all the papers in the dental field, only three models include the reproduction of the entire skull combining photoelastic materials with other non-photoelastic resins or with natural bony structures. The periodontal ligament was taken into account as a separate structure in only a few of the reviewed articles. This tissue has an important biomechanical role absorbing loads during chewing; it would therefore be important to study the possibility of including it in the models with some material that resembles its properties. In contrast, in the medical field, anatomical considerations were better replicated in the models; from the simplest ones that consider the bone shape in only two dimensions [93] to the reproduction of complete joints such as the knee [91]. Very complete models were developed for some theses [116,117,118]. Chrcanovic [119] in his review mentions that to be able to better suggest reality with photoelasticity the complete anatomical shape should be taken into account and reproduced. A wide variety of models have been used, but there is room for improvement. Even if difficult, models need to be developed according to the needs of each area and should include all the structures involved so that they are closer to reality.

Rigid or flexible epoxy resins are generally used. Few authors consider the properties of bones, teeth and ligaments; when they do so they combine materials [84]. Some resins have properties similar to bone or teeth; when choosing the resin with which to make a model it is necessary to find a balance between those that allow a correct visualization of the optical phenomenon and consistency. Resins that are more flexible are more sensitive to light but do not reflect the characteristics of all the tissues intended to replicate. It is necessary to consider alternatives of the resins to use since the most frequently mentioned resins are not always available or commercialized in all countries.

While photoelasticity has been used in research it has not been fully taken advantage of for teaching. In other areas such as engineering, the use of this technique as a teaching tool has already been validated by demonstrating a better understanding by the students of the physical phenomena of stress distribution. Pérez et al. [120] described “solid mechanics” to students using photoelasticity. They questioned them on how useful the technique was for understanding this complex subject; 91% mentioned the technique as useful and 88% indicated that they would consider using this technique again for other issues in solid mechanics. There were no reports of its use as a didactic tool at either the undergraduate or graduate level in the health areas. It is easy to imagine that if the use of photoelasticity improves the understanding of the concepts and facilitates their application in engineering students for whom the theory of stress distribution is a familiar concept, dental and medical students would greatly benefit from the introduction of biomechanical concepts in a much more user-friendly way and making the most of these concepts in the clinical field. Dental and medical schools should, therefore, include photoelastic models to illustrate the biomechanical functioning of biological systems. Improving students’ awareness of the impact of treatments on bone, joint, dental and periodontal structures with these models could reduce the number of unfavorable circumstances that may arise from the unawareness of stress distribution on the tissues.

Limitations of this study were not reporting the complete methodology in the tables to compare variations in the way the models were obtained, or if photoelasticity was complemented by some other method, to try to determine which ones work better; however, in that case, we would have had to communicate with several authors to obtain missing data. Since we did not include the studies prior to 2000 we may have missed studies that could have mentioned the use of photoelasticity for teaching. Although this project was limited to inquiring into the applications of photoelasticity in the medical and dental area and only takes into account other techniques for comparative purposes, the possibility of combining photoelasticity with other techniques and in other areas of health could later be analyzed. Therefore, an in-depth analysis of the models and materials used to apply this method could be carried out and information could be obtained on which materials provide better observation results and image quality and thus obtain useful quantitative data for teaching.

More research is needed to better support the information that photoelasticity can provide to research in the health areas, but there is great potential in its use in didactics. While it is true that the technique has several limitations, these limitations are not truly problematical for its use in teaching, as long as models that are closer to clinical reality can be developed. Other available techniques to describe the same phenomena in biomechanics, are more complex and their cost make them unfeasible to be used in classrooms.

## 5. Conclusions

In both medicine and dentistry, the use of the photoelastic method has been underused and limited to a few specialties; however, in the medical field, the models used are more complex and not limited to hard tissues. In dentistry, only specialties related to implantology have clearly taken advantage of its possibilities.

Photoelastic models are an alternative towards the understanding of biomechanics in the area of the health sciences; they visually provide qualitative information on the extension, origin and direction of stress. The objectives reported in the documents found were mainly for research and some simplify the geometry in order to have fewer errors in the calculations and because of this they do not faithfully copy the complex anatomy of the stomatognathic or human systems. Few models have taken the different anatomical characteristics and types of tissues involved into account which are essential to better emulate human biomechanics; further improvements are therefore required.

It is important to highlight that, despite the fact that photoelastic models have been used since the 1970s, especially in the engineering area, we did not find any evidence on their use as a material to assist education related to biomechanics in medicine or dentistry. Therefore, there are many areas of opportunity for the use of this technique in research to better explain biomechanical behavior, but even more so to simplify and support teaching biomechanics in an affordable and straightforward way to both dentists and doctors in training.

## Figures and Tables

**Table 1:** Characteristics and findings of studies on implants for fixed prostheses in the dental area.

Reference	Objective	Type of Model and Photoelastic Material Used	Data
Gross et al., 2001 [23] J. Prosthet. Dent. Israel	To model the stress distribution around maxillary implants comparing occlusal loading in a two-dimensional photoelastic model and a dry skull.	Reproduction of a skull cut at the level of the 1st molar and a dry skull covered with photoelastic film. PS-2A, “K” factor, PCI, PL8, Vishay Measurements Group Inc., Raleigh, NC, USA.	Stress distribution of maxillary loads around first molar implants.
Guichet et al., 2002 [24] J. Prosthet. Dent. USA	To examine the effect of splinting and interproximal contact tightness on passivity of fit and the load transfer characteristics of implant restorations.	Reproduction of a partially edentulous lower left quadrant with fixed prosthesis and implants included in photoelastic resin. PL 2 Vishay Measurements Group Inc., Malvern, PA, USA.	Stress with an increase in contact tightness.
**Ochiai et al., 2003 [25] J. Prosthet. Dent. USA**	To compare stress transfer patterns with 1 or 2 posterior implants connected to single anterior simulated natural teeth with 1 or 2 implant abutments under functional loads.	Reproduction of a partially edentulous arch with implants included in photo-elastic resin. PL-2 Vishay Measurements Group Inc., Raleigh, NC, USA. Teeth in photoelastic resin with roots. PLM-1 Vishay Measurements Group Inc., Raleigh, NC, USA. Periodontal ligament. Solithane Uniroyal Chemical, Co Inc., Middlebury, VT, USA	Stress levels with prostheses supported by 2 implants and a tooth.
Ueda et al., 2004 [26] Brazilian Oral Res. Brazil	To compare the stress distribution in a fixed prosthesis with 3-parallel implants, to the same prosthesis with an angled central implant.	Implants included in a resin mold. PL 2 Vishay Measurements Group Inc., Raleigh, NC, USA.	Influence of the angulation or parallelism of implants supporting a 3 unit fixed prosthesis.
Araújo et al., 2009 [27] IFMBE Proceedings Brazil	To compare the effect of varying the type of interproximal contact in fixed partial dentures over three adjacent implants.	Implants included in a resin cube with roots. Flexible Polipox Ind. E. Com. Ltd.a., Sao Paulo, Brazil.	Shear stress values of different crown designs and contact points for supported implant prosthesis.
Odo et al., 2010 [28] Braz. Dent. Sci. Brazil	To evaluate five transfer techniques in osseointegrated implants.	Implants included in a photoelastic resin cube. Araldite^®^ GY 279 by Huntsman, Brazil.	Stress distribution on implants generated by different impression techniques.
De Torres et al., 2011 [29] J. Biomech Brazil	To compare stresses transmitted to the implants from the metal framework and to investigate a possible correlation between vertical misfits and these stresses.	Implants included in a semi-hexagonal prism of photoelastic resin. Polipox, Sao Paulo, Brazil.	Stress transmitted to implants from frameworks of different materials and vertical mismatch.
Castro et al., 2012 [30] Odonto. Brazil	To evaluate the influence of laser welding or TIG welding of cylindrical rods of cobalt-chromium in the generation of tensions around implants.	Implants included in a resin mold. Araldite^®^ GY 279 by Huntsman, Brazil.	Stress distribution around implants joined by different welding techniques.
Zielak et al., 2013 [31] RSBO Brazil	Colorimetric photoelastic analysis of tension distribution around dental implants under axial loads.	Implants included in a resin mold. Flexible epoxy resin, Glll Polipox, Sao Paulo, Brazil.	Correlation of some implant characteristics to the areas of stress distribution.
Pellizzer et al., 2013 [32] Rev. Cir. Traumatol. Buco-Maxilo-Fac. Brazil	To evaluate the influence of increasing the length of the implant around the body of the implant.	Implants included in a resin mold. PL 2 Vishay Measurements Group Inc., Malvern, PA, USA.	Stress caused by increasing the length of the implant.
Aguiar et al., 2013 [33] J. Appl. Oral Sci. Brazil	To evaluate the importance of a distal proximal contact on the load transfer to the posterior region of the mandible by non-splinted adjacent implant-supported crowns.	Teeth and implants included in a resin mold. Araldite^®^ GY 279 by Huntsman, Brazil.	Importance of distal contacts for load distribution in implant-supported fixed prostheses.
De Castro et al., 2013 [34] Braz Dent. J. Brazil	To evaluate stress distribution on implants while changing passivity with three different processes of manufacturing metal frameworks.	Implants included in a resin mold. Flexible epoxy resin Glll Polipox, Sao Paulo, Brazil.	Stress distribution changing passivity of Co-Cr implants with different manufacturing processes of the metal framework.
Cidade et al., 2014 [35] Braz. Oral Res. Brazil	To evaluate two different angulations of the cantilever in fixed implant-supported maxillary complete dentures.	Implants included in a resin mold arch. Flexible silicone and ASB-10Blue GlV Polipox, Sao Paulo, Brazil.	Stress distribution around different implant angulations and loading areas.
Emidio et al., 2014 [36] Odonto. Brazil	To evaluate the peri-implant stress with and without the use of a flat occlusal splint under various loads.	Implant included in a rectangular resin block. Polipox photoelastic resin Ind. E. Com. Ltd.a., Sao Paulo, Brazil.	Peri-implant stress with and without the use of a flat occlusal splint.
Tiossi et al., 2014 [37] J. Prosthet. Dent. Brazil	To compare the photoelasticity and digital image correlation to analyze the stresses/strains transferred by an implant-supported prosthesis.	Implants included in a photoelastic resin block with teeth and a 2 unit fixed prosthesis. Araldite^®^ GY 279 by Huntsman, Brazil.	A comparison of photoelasticity and digital imaging techniques to demonstrate stress/strain.
Cruvinel et al., 2015 [38] Mater. Res. Brazil	Manufacture a new zirconia implant and to evaluate the fracture strength and stresses generated after cyclic loading comparing it to titanium implants.	Implants included in a resin cube. Resin not specified.	Stress distribution in Zr and Ti implants after cyclic loading.
Lencioni et al., 2015 [39] J. Prosthet. Dent. Brazil	To evaluate the vertical misfit, passivity, and biomechanical behavior of a prosthetic protocol with an I-shaped beam framework.	Implants included in a resin mold arch. Araldite^®^ by Huntsman, Brazil.	Stress distribution around implants with a 1-piece cast or laser welded I-shaped beam framework.
De Moraes et al., 2015 [40] Int. J. Odontostomat. Brazil	To analyze the stress distribution in craniofacial structures around zygomatic implants.	Implants in a reproduction of the zygomatic and maxillary bone with photoelastic resin. Polipox lll flexible epoxy resin, Sao Paulo, Brazil.	Stress distribution in craniofacial structures around zygomatic implants.
De Araujo et al., 2015 [41] Braz. Dent. J. Brazil	To evaluate the passivity of frameworks screwed on abutments by measuring the passive fit and strain development, and to compare CAD/CAM technology with samples manufactured by conventional casting.	Implants included in a resin cube. Flexible Polipox Ind. E. Com. Ltd.a., Sao Paulo, Brazil.	Relative passivity of dental implants manufactured with CAD-CAM.
Zielak et al., 2015 [42] Res. Biomed. Eng. Brazil	To analyze the biomechanics of two different types of short implants under axial loads.	Implants embedded in a resin mold. GIII Flexible Epoxy Photoelastic, Polipox, Sao Paulo, SP, Brazil.	Stress distribution generated by the influence of the design of the implant.
Lee et al., 2016 [43] J. Prosthet. Dent. Korea	To compare the stress distribution in the supporting tissues surrounding implants placed in the anterior maxilla with 5 partial fixed dental prosthetic designs.	Reproduction of an upper arch with premolars, 6 anterior implants included in a photoelastic resin and prosthetic rehabilitation. PL 2 Vishay Measurements Group Inc., Malvern, PA, USA.	Stress distribution with different partial fixed prosthetic designs.
Presotto et al., 2017 [44] J. Prosthet Dent. Brazil	To evaluate the effect of the prosthetic framework fabrication method on the marginal fit and stress transmitted to implants.	Implants included in a resin mold with a prosthetic framework. Araldite^®^ GY 279 by Huntsman, Brazil.	Stress distribution of a 3 unit prosthesis manufactured by CAD-CAM and overcasting.
Presotto et al., 2018 [45] BJOS Brazil	To compare the reliability between photoelastic and finite element (FE) analyses by evaluating the effect of different marginal misfit levels using conventional and short implants.	Implants included in a resin cube with a fixed 3 unit prosthesis. Araldite^®^ GY 279 by Huntsman, Brazil.	Tensions generated by marginal mismatch in implant-supported prosthesis.

References in bold indicate studies that used more complete models.

**Table 2:** Characteristics and findings of studies on implants for total removable prostheses in the dental area.

Reference	Objective	Type of Model and Photoelastic Material Used	Data
Sadowsky et al., 2000 [46] J. Prosthet. Dent. USA	To compare the load transfer of different simulated mandibular-retained overdenture designs on multiple implants.	Reproduction of an edentulous jaw with implants in photoelastic resin and an overdenture. PL-2, Photoelastic Division, Vishay Measurements Group Inc., Raleigh, NC, USA.	Stress distribution of a mandibular overdenture.
Ochiai et al., 2004 [47] J. Prosthet. Dent. USA	To evaluate the effect of palatal support on load transfer for 3 maxillary implant-supported overdenture designs.	Implants included in a toothless resin maxillary arch. PLM-2 Vishay Measurements Group Inc., Raleigh, NC, USA.	Stress distribution around implants with and without palatal coverage.
Sadowsky et al., 2004 [48] J. Prosthet. Dent. USA	To compare the load transfer of different simulated mandibular cantilever bar–retained prostheses on both a 2-implant and a 3-implant design.	Reproduction of an edentulous jaw with implants included in photoelastic resin and a total prosthesis. PL 2 Photoelastic Division, Vishay Measurements Group Inc., Raleigh, NC, USA.	Stress distribution around implants that hold dentures with clips.
Celik et al., 2007 [49] J. Prosthet. Dent. Turkey	To compare the load transfer of 4 mandibular fixation systems with 3 vertically inclined implants.	Dental arch reproduction with implants included in photoelastic resin. PL 2 Vishay Measurements Group Inc., Malvern, PA, USA.	Stress distribution generated by implants placed at different angles, as well as different designs to support removable total prosthesis.
**Pigozzo 2010 [50]** **Rev. Odontol. Univ. Cid. São Paulo. Brazil**	To appraise the load transmission in bar-clip retention systems for overdentures with 2 simulated implant positions.	Reproduction of a skull with teeth and implants included in a photoelastic material. PL 2 Vishay Measurements Group Inc., Malvern, PA, USA.	Stress distribution around the implants sustaining the support bar for the removable total prosthesis.
Asvanud et al., 2011 [51] J. Prosthet. Dent. USA	To compare the load transfer of a complete-arch restoration supported by 4 implants with external and internal implant–abutment connections.	Implants included in a resin arch with over dentures. PL4-M Vishay Measurements Group Inc., Raleigh, NC, USA.	Stress distribution around dental implants with internal vs. external connections to fix total prostheses to implants.
**Pigozzo et al., 2013** [52] **Brazilian Oral Res.** **Brazil**	To evaluate the stress distribution in mandibular bone surrounding a bar-clip overdenture with 2 simulated implant angulations.	Reproduction of a skull with teeth and implants included in a photoelastic material. PL 2 Vishay Measurements Group Inc., Malvern, PA, USA.	Stress distribution around implants in the jaw to support total removable prosthesis.
Cidade et al., 2015 [53] Int. J. Odontostomat. Brazil	To evaluate the load distribution in tilted distal implants used in the all-on four system.	Implants included in a replica of an edentulous jaw. Araldite^®^ GY 279 by Huntsman, Brazil	Stress distribution caused by inclination of implants for the rehabilitation with the all-on-four model.
Pereira et al., 2015 [54] Mater. Sci. Eng. C Brazil	To evaluate the stresses induced on the alveolar bone ridge by lined conventional complete mandibular dentures.	Reproduction of edentulous models and total prostheses in photoelastic resin. Araldite^®^ GY 279 by Huntsman, Brazil.	Tensions transmitted by removable total prostheses in maximum intercuspidation.
Ramesh et al., 2016 [55] Opt. Lasers Eng. India	To analyze the uses of digital photoelasticity to propose a new 3D model and method of data analysis.	Reproduction of an edentulous lower arch with implants included in a photoelastic resin. Araldite^®^ CY230 by Huntsman, Brazil.	A novel interpretation of data obtained by photoelasticity of stress distribution around dental implants.
Pimentel et al., 2017 [56] Brazilian Oral Res. Brazil	To evaluate the stress behavior around short implants in edentulous atrophic mandibles.	Implants included in a resin mold, reproducing the mandibular position. GlV Polipox flexible epoxy resin, Sao Paulo, Brazil.	Stress generated by mandibular implants according to length, width and geometry.
Zaparolli et al., 2017 [57] Mater. Sci. Eng. C Brazil	To compare the stress distribution of mandibular full dentures supported with implants given the material and manufacturing of the bar.	Reproduction of a mandibular arch with implants included in a photoelastic resin and removable total prosthesis. Araldite^®^ GY 279 by Huntsman, Brazil.	Stress distribution around an implant varying the material and manufacturing technique of the removable total prosthesis.
Campaner et al., 2019 [58] J. Clin. Diagnostic Res. Brazil	To evaluate the biomechanical behavior of overdentures supported by 1 or 2 implants with different types of connectors and submitted to compression.	Reproduction of the lower jaw with implants included in a photoelastic resin and total prosthesis. PL-2, Vishay Measurements Group Inc., Raleigh, NC, USA.	Biomechanical behavior of total prostheses varying the number of implants that support it.

References in bold indicate studies that used more complete models.

**Table 3:** Characteristics and findings of studies on orthodontics in the dental area.

Reference	Objective	Type of Model and Photoelastic Material Used	Data
Dobranszky et al., 2009 [59] Rev. Dent. Press. Ortodon. Ortop. Facial Brazil	To study the area where the force is exerted after the activation of orthodontics devices.	Teeth joined by orthodontic appliances with roots embedded in transparent jelly. Oetker ™ transparent jelly and glycerin.	Stress generated by movements in orthodontics.
Maia et al., 2010 [60] Dental Press. J. Orthod. Brazil	To evaluate the force system produced by the T-spring used for space closure.	Teeth included in a resin mold. Flexible epoxy resin CMR-201 Polipox, Sao Paulo, Brazil.	Force generated by the T-spring system with different pre-activations.
Dobranszky et al., 2010 [61] Rev. Mater. Brazil	To evaluate the stress distribution in the resin in contact with the screw string of cylindrical and conical mini-implants.	Mini implants included in a photoelastic material. Transparent jelly and glycerin.	Tension analysis by lateral load in mini orthodontic implants with different geometries.
Maia et al., 2011 [62] Dental Press. J. Orthod. Brazil	To evaluate the force system generated by T-springs placed in the interbracket space using the pre-activation advocated by Burstone.	Teeth included in a resin mold. Flexible epoxy resin CMR-201 Polipox, Sao Paulo, Brazil.	Force generated in a model with two canines by a pre-activated orthodontic spring.
Claro et al., 2011 [63] Dental Press. J. Orthod. Brazil	To study stress distribution generated by Rickett’s utility arch in a photoelastic model.	Teeth included in a resin mold. Gll Polipox epoxy resin, Sao Paulo, Brazil.	Stress distribution in the intrusion zone of mandibular incisors using Rickett’s utility arch.
Claro et al., 2014 [64] Dental Press. J. Orthod. Brazil	To compare dental and skeletal anchorages in mandibular canine retraction by stress distribution analysis.	Teeth included in a resin mold. Flexible epoxy resin Glll Polipox, Sao Paulo, Brazil.	Stress generated during canine retraction.
Sobral et al., 2014 [65] Dental Press. J. Orthod. Brazil	To analyze the stress caused by conventional and self-ligating brackets with expanded arch wires.	Teeth included in a resin mold. Polipox flexible epoxy resin, Sao Paulo, Brazil.	Wire-generated stress by conventional and self-ligating brackets.
Portes et al., 2017 [66] ReBraM Brazil	To evaluate stress distribution after the insertion of mini orthodontic implants of two different brands.	Mini orthodontic implants included in rectangular resin molds. Araldite^®^ GY 279 by Huntsman, Brazil.	Stress generated around mini orthodontic implants of different brands and designs.
Schwertner et al., 2017 [67] Dental Press. J. Orthod. Paraguay	To evaluate the effects generated by the Connecticut Intrusion Arch.	Upper anterior teeth and first molars included in a resin mold. GlV Polipox flexible epoxy resin, Sao Paulo, Brazil.	Stress distribution generated by the Connecticut Intrusion Arch.
Abrao et al., 2018 [19] AJO-DO Brazil	To analyze and compare the stress distribution with different molar uprighting techniques.	Resin arch with canines, premolars and 1st molars. Flexible epoxy resin Epoxi Glass Diadema, Brazil.	Analysis of different orthodontic treatments to solve mesial inclination.

**Table 4:** Characteristics and findings of studies on implants for surgery in the dental area.

Reference	Objective	Type of Model and Photoelastic Material Used	Data
Cebrián et al., 2012 [68] Rev. Esp. Cir. Oral Maxilofac. Spain	To develop a biomechanical simulator of the masticatory skeletal muscle system.	Mini-implants included in a photoelastic resin jaw. Epoxy resin.	Use of 3D photoelasticity to evaluate stress distribution in a biomechanical model.
Andrade et al., 2014 [69] Int. J. Odontostomat. Brazil	To identify the stresses produced by osteosynthesis screws in the fixation of a sagittal osteotomy of the mandibular ramus.	Implants included in a reproduction of a mandible with osteotomy. Araldite^®^ by Huntsman, Brazil.	Tensions generated by osteosynthesis screws.
Falci et al., 2014 [70] Oral Surg. Oral Med. Oral Pathol. Oral Radiol. Brazil	To compare the performance of cannulated screws with other fixation methods in fractures of the mandibular symphysis.	Photoelastic resin jaw with a fracture. Araldite^®^ GY 279 by Huntsman, Brazil.	Stress distribution generated by fixation methods for mandibular fractures.
Rodrigues et al., 2015 [71] J. Craniomaxilofac. Surg. Brazil	To compare four methods of fixation for fractures of the mandibular body.	Reproduction of an hemimandible in photoelastic resin. Araldite^®^ GY 279 by Huntsman, Brazil.	Stress in the bone generated by dental fixation elements in jaw fractures.
De Lima et al., 2015 [72] Oral Maxillofac. Surg. Brazil	To compare the performance of cannulated screws vs. solid-core screws.	Polyurethane jaws embedded in transparent mineral oil.	Stress distribution in the mandible by fixation screws.
Araújo et al., 2015 [73] Oral Maxillofac. Surg. Brazil	To analyze the hardness and residual stress in the regions of the fixing plate by manually or prefabricated bends.	Pre-folded maxillary fixation element included in a resin. Epomet Molding Compound^®^ Buehler.	Residual stress produced by either manually or prefabricated bending of the maxillary fixation elements.

**Table 5:** Characteristics and findings of studies on operative dentistry.

Reference	Objective	Type of Model and Photoelastic Material Used	Data
**Butzke et al., 2007** [74] **Acta Odontol. Venez. Brazil**	To evaluate the distribution and concentration of tension in the bone that supports the upper premolars.	Tooth cuts reproduced with photoelastic resin on a polystyrene support simulating the alveolar bone and the periodontal ligament simulated with IMPREGUM F (3M-ESPE). Non specified.	Stress distribution of the masticatory loads generated in the alveolar bone with different dental cavities and restorations.
Lopes et al., 2008 [75] J. Appl. Oral Sci. Brazil	To evaluate polymerization shrinkage and shrinkage stress of composites polymerized with a LED and a quartz tungsten halogen light source.	Class I dental cavity replications in photoelastic resin molds. Crystal 2120 Redelease transparent epoxy resin, Sao Paulo, Brazil.	Polymerization shrinkage reflected in cavity walls.
Lopes et al., 2011 [76] Brazilian Oral Res. Brazil	To evaluate the polymerization stress generated by a silorane-based composite.	Photoelastic resin molds with cavities and composite. Flexible epoxy resin Glll Polipox, Sao Paulo, Brazil.	Stress generated in the wall of a cavity filled with a silorane-based resin.
Oliveira et al., 2012 [77] Brazilian Oral Res. Brazil	To compare polymerization stress in composites made with camphorquinone and/or phenylpropanedione as photoinitiators.	Photoelastic resin discs with cylindrical cavities. Araldite^®^ GY 279 by Huntsman, Brazil.	Stress in cavity walls due to contraction during polymerization.
Oliveira et al., 2012 [78] Acta Odontol Latinoam Brazil	To evaluate the polymerization stress and degree of conversion of a composite submitted to different photoactivation protocols.	Photo elastic resin molds with cavities. Araldite^®^ GY 279 by Huntsman, Brazil.	Stress distribution in cavity walls comparing different photoactivation protocols.
Pereira et al., 2018 [79] Clin. Cosmet. Investig. Dent. Brazil	To evaluate the influence of increments in thickness on degree of conversion, Knoop microhardness, and polymerization-shrinkage stress of three dental composites.	Maxillary second premolar models with a standard class I cavity and restoration. Epoxy resin flexible GIV; Polipox, Cesário Lange, SP, Brazil.	Stress distribution and degree of conversion by polymerization of dental composites.

References in bold indicate studies that used more complete models.

**Table 6:** Characteristics and findings of studies on occlusion in the dental area.

Reference	Objective	Type of Model and Photoelastic Material Used	Data
**Cebrián et al., 2009** [18] **Rev. Esp. Cir. Oral y Maxilofac. Spain**	To develop a 3D static simulator of the masticatory system to analyze stress distribution in various physiological and pathological situations.	A mandibular replica in a photoelastic resin articulated to a skull. Epoxy resin.	Stress distribution of the musculoskeletal system in physiological and pathological situations by tension-freezing.
Yamamoto et al., 2012 [80] J. Biomech Japan	To detect the direction of bite force to establish a quick clinical method before the placement of a dental implant.	Bite registration in photoelastic material. Ethyl vinyl acetate, cellulose acetate and silicone.	Stress distribution produced by occlusal loads through stress freezing.
Judge et al., 2003 [81] J. Prosthet. Dent. Australia	To describe an indirect technique to evaluate the redistribution of loads generated during simulated mastication.	Dog skull where external plates of photoelastic resin overlap. PL-8; Photoelastic Division, Vishay Measurements Group Inc., Raleigh, NC, USA.	Chewing stress distribution observed by an indirect technique.

References in bold indicate studies that used more complete models.

**Table 7:** Characteristics and findings of implant studies for maxillofacial prostheses in the dental area.

Reference	Objective	Type of Model and Photoelastic Material Used	Data
**Lyons et al., 2005** [82] **J. Prosthet. Dent. USA**	To compare the forces exerted on the supporting structures of abutment teeth in different sizes and surgical resections of removable partial prosthetic designs.	Teeth with roots included in a reproduction of maxillary defects PLM-1 Vishay Measurements Group Inc., Raleigh, NC, USA. Periodontal ligament Solithane Uniroyal Chemical, Co Inc. and a complete or half arch in photoelastic resin. PL 2 Vishay Measurements Group Inc., Malvern, PA, USA.	Stress generated by partial obturators in different types of maxillectomy.
Gioato et al., 2017 [83] J. Prosthet. Dent. Brazil	To evaluate dissipation loads applying shear forces to different attachment systems used with implant-retained obturators.	Reproduction of an arch with implants included in photoelastic resin with mounted dentures. PL 2 Vishay Measurements Group Inc., Raleigh, NC, USA.	Stress distribution around implants that support maxillary obturators.

**Table 8:** Characteristics and findings of studies on **removable partial dentures** in the dental area.

Reference	Objective	Type of Model and Photoelastic Material Used	Data
**Thompson et al., 2004** [84] **J. Prosthet. Dent. USA**	To compare the forces exerted on the supporting structures of the abutment teeth by seven removable partial denture designs using a photoelastic model.	Reproduction of a partially edentulous lower arch in photoelastic resin with removable prosthesis. Teeth and roots PLM-1Z, Vishay Measurements Group Inc., Malvern, PA USA. Ligament Solithane, thiokol chemical Co. Bone base PL 2 Photoelastic Inc. Malvern, PA, USA.	Stress distribution of occlusal loads according to the design of the supports of removable partial dentures.
**Pellizer et al., 2010** [85] **Acta Odontol Latinoam** **Brazil**	To analyze the distribution of stress caused by four types of removable partial denture designs on four different types of residual ridges.	Reproduction of residual ridges and anterior teeth with roots included in a photoelastic resin. Teeth PL 1 Vishay Measurements Group Inc., Raleigh, North Carolina USA. Bone PL 2 Vishay Measurements Group Inc., Raleigh, NC, USA.	Tensions generated by different removable partial dentures on different types of residual ridges.

References in bold indicate studies that used more complete models.

**Table 9:** Characteristics and findings of studies on **endodontics** in the dental area.

Reference	Objective	Type of Model and Photoelastic Material Used	Data
Bosso et al., 2015 [86] Braz Dent. J. Brazil	To quantify and evaluate the distribution of stress in the root produced by different endodontic posts.	Reproduction of teeth in photoelastic resin. Flexible epoxy resin Gll Polipox, Sao Paulo, Brazil.	Intra-radicular stress of different types of endodontic posts.
Viela et al., 2016 [87] Braz. Dent. Sci. Brazil	To perform an in vitro analysis of the stress related to instrumentation of artificial root canals with the Reciproc System.	Resin cubes with simulated root canals. Transparent epoxy resin.	Stress analysis generated by endodontic instrumentation.

**Table 10:** Characteristics and findings of studies on basic science in the dental area.

Reference	Objective	Type of Model and Photoelastic Material Used	Data
Sui et al., 2014 [88] Acta Biomater. UK	To determine the internal lattice strain response of human enamel samples as a function of in situ uniaxial compressive loading.	Enamel samples included in photoelastic resin. Epoxy resin, Buehler Epokwick, ITW Test & Measurement GmbH.	Analysis of the mechanical properties of human enamel.

## Data Availability

Not applicable.
